# How can the risk of ovarian retorsion be reduced?

**DOI:** 10.1186/s13256-018-1677-0

**Published:** 2018-07-04

**Authors:** Feride Mehmetoğlu

**Affiliations:** Department of Pediatric Surgery, Dörtçelik Children’s Hospital, 16140 Bursa, Turkey

**Keywords:** Child, Ovarian torsion, Detorsion, Retorsion, Oophorectomy, Oophoropexy

## Abstract

**Background:**

In the current treatment of idiopathic ovarian torsion, the use of oophorectomy has declined in favor of preserving the ovary. This approach brings with it the question of how to reduce the possibility of retorsion of the detorsioned ovary. The aim of this study was to analyze how retorsion can be prevented.

**Methods:**

Five patients (a 30-day-old Caucasian girl, a 55-day-old Caucasian girl, an 8-year-old Caucasian girl, a 10-year-old Caucasian girl, and a 16-year-old Caucasian girl) who underwent surgery due to non-neoplastic ovarian torsion were retrospectively analyzed for diagnosis and treatment in terms of reducing the possibility of retorsion.

**Results:**

In all patients, a precise diagnosis of idiopathic unilateral ovarian torsion was made during laparotomy, and the patients underwent different procedures. The ovary was found to be autoamputated in one patient, and two patients underwent salpingo-oophorectomies due to adnexal necrosis. The ovaries were detorsioned in the remaining two patients. During the operations, patients were evaluated regarding the prevention of retorsion of the ipsilateral and/or contralateral ovary; cyst drainage, cystectomy, ligament fixation, and/or oophoropexy were performed. The median follow-up period of the patients was 2 years (range 1.5–6 years), and they continue to be followed uneventfully.

**Conclusions:**

To date, there is no standard approach to protect the ovary from retorsion in patients who undergo surgery due to torsion. The surgical procedure should be tailored on a case-by-case basis.

## Background

Although ovarian torsion is uncommon, early diagnosis and treatment are important, especially during childhood, as torsion can otherwise result in organ loss, adversely affect pubertal development, and cause infertility. Ovarian torsion is usually diagnosed late due to the lack of specific clinical findings and imaging methods [1]. Torsions often occur on one side and only once; however, recurrence in the same ovary or both ovaries is possible. Ovarian torsions are generally considered the result of a sudden increase in ovarian volume due to cysts, masses, or excessive mobilization due to a long mesosalpinx. However, most torsioned ovaries have been reported to be normal [1, 2].

Over the past two decades, significant changes have occurred in the approach to ovarian torsion. Oophorectomy was frequently performed in these cases due to the fear of leaving necrotic ovary tissue, suspicion of malignancy, and the risk of pulmonary embolism and peritonitis [3]. Most researchers now believe that future hormonal activity is possible and thus suggest ovarian detorsion, followed by leaving the ovary in place and evaluating the possibility of subsequent oophoropexy [4, 5].

This conservative treatment approach has brought with it the question of how to reduce the possibility of retorsion of the detorsioned ovary. In contrast to testicular torsion, for ovarian torsion, no consensus exists in the literature regarding methods to prevent retorsion [6]. Classically, upper pole excision, cystectomy, and cyst aspiration have been used for this purpose. Current discussion revolves around how the process should be performed, with debate regarding the advantages and disadvantages of fixation methods on the same or the opposite side of the detorsioned ovary. A review of the literature reveals that no standard approach has been established.

The aim of the present study was to determine the surgical techniques that can be performed to prevent retorsion and protect the ovaries in patients who undergo surgery due to idiopathic ovarian torsion.

## Methods

The hospital records of patients diagnosed as having non-neoplastic ovarian torsion from November 2010 to May 2017 were reviewed retrospectively. Five patients who underwent different procedures were selected. The demographic features and age of the patients, onset of symptoms, clinical findings, radiological findings, operative methods, anatomical and pathological features of the ovaries observed during surgery, procedures performed, histopathology, and late radiological follow-up were evaluated. The findings were examined with respect to the existing literature. All patients operated on and followed up by the same pediatric surgeon were either admitted to the emergency unit or pediatric surgery out-patient clinic in Bursa Dörtçelik Children’s Hospital, Turkey. This study has all of the recognized limitations of a retrospective case series.

## Results

The operative ages of the five patients diagnosed as having non-neoplastic ovarian torsion were 30 days, 55 days, 8 years, 10 years, and 16 years. The two cases of babies (cases 1 and 2) were referred to us by a gynecologist who had detected an intraabdominal cystic mass during prenatal ultrasonography (US) in the last trimester, and the three adolescent patients (cases 3, 4, and 5) were admitted with serious acute pelvic pain. No other factors were present in the patients’ histories and all patients were otherwise healthy. The demographic features and clinical findings of the patients are summarized in Table 1.

**Table 1:** Summary of clinical history, imaging and operative data

CASES	Case 1	Case 2	Case 3	Case 4	Case 5
Age	30 days	55 days	8 years	10 years	16 years
Menarche	–	–	–	–	+
Complaint	Prenatal imaging	Prenatal imaging	Acute abdominal symptoms	Acute abdominal symptoms	Acute abdominal symptoms
Imaging	Prenatal: US, Postnatal: X-ray, US, Doppler US, CT	Prenatal: US, Postnatal: X-ray, US, Doppler US, CT	–	–	US
Side	Left	Right	Right	Left	Right
Detorsion	–	–	–	Ipsilateral	Ipsilateral
Oophorectomy	S. oophorectomy	Autoamputation	S. oophorectomy	–	–
Cyst aspiration	Contralateral	Contralateral	–	–	Contralateral
Cystectomy	–	–	–	–	Ipsilateral
Oophoropexy	Contralateral	–	–	–	–
Ligament fixation	–	–	–	Ipsilateral	–

*CT* computed tomography, *S.* salpingo, *US* ultrasonography

During the operations, five patients with a diagnosis of idiopathic unilateral ovarian torsion were evaluated regarding the prevention of retorsion of the ipsilateral and/or contralateral ovary.

None of the patients developed wound infections or peritonitis during the postoperative (PO) period, and the patients who were fed on the first PO day were discharged on the second to fifth PO days. Ovaries were checked regularly with pelvic and Doppler imaging after surgery. The median follow-up period of the patients was 2 years (range 1.5–6 years), and they continue to be followed uneventfully.

## Case presentations

### Case 1

**Fig. 1:**
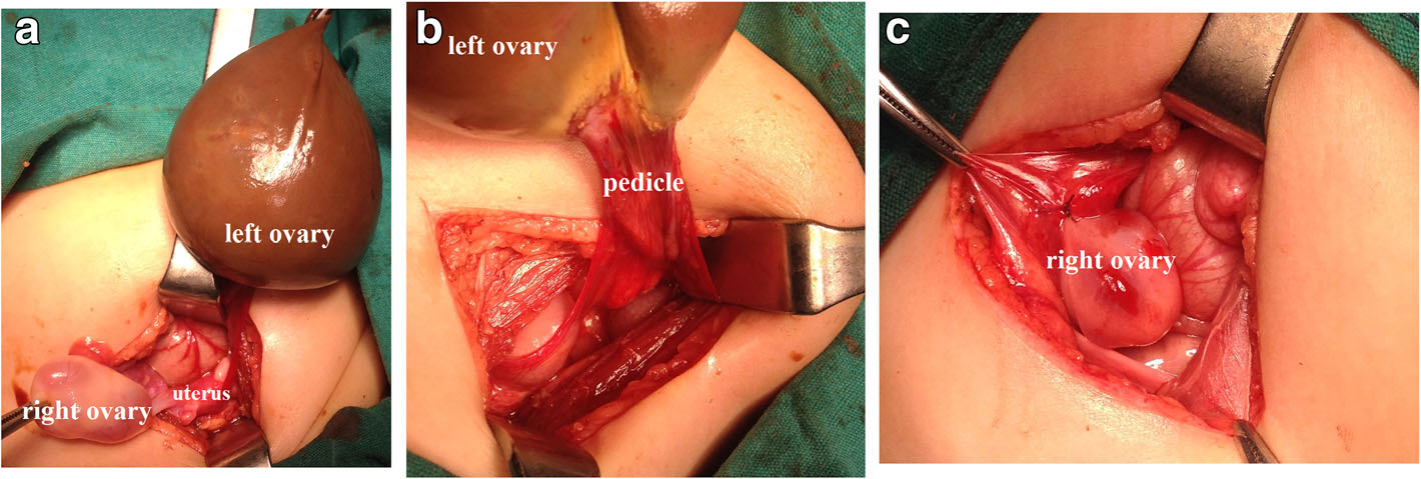
**a** Left necrotic ovary, infantile uterus, and right ovary with cysts. **b** Left adnexa lacking normal ovarian and pedicle tissues. **c** Remaining right ovary fixed to the sidewall of the abdomen

A 30-day-old Caucasian baby girl was referred to our hospital by a gynecologist who had detected an intraabdominal cystic mass during prenatal US in the last trimester. A physical examination revealed a mass of approximately 6 cm in diameter that could be palpated in the midline below the umbilicus. No other factors were present in her history. Imaging studies showed intraabdominal cystic mass. The tumor markers that were examined to determine the presence of malignancy were within normal ranges. During surgery, torsion was detected in her left adnexa; a left cystic mass with torsion was necrotic in appearance and was completely lacking normal ovarian and fallopian tube tissue. A left salpingo-oophorectomy was performed. An oophoropexy was performed on her right ovary with absorbable suture at the level of the pelvic brim of the sidewall of her abdomen after draining peripheral cysts (Fig. 1a–c); an incidental appendectomy was performed. Pathologic examination of the specimen confirmed the diagnosis of a necrotic ovary. She had cysts smaller than 1 cm on the existing single contralateral ovary they were aspirated during surgery. Postoperatively the cysts redeveloped up to 2 cm and spontaneously disappeared after 6 months of follow-up. She developed no wound infections or peritonitis during the PO period and was discharged on the third PO day. Her ovary was checked regularly with pelvic and Doppler imaging after surgery; the development of her ovary was also age appropriate. She has been followed for 2 years.

### Case 2

**Fig. 2:**
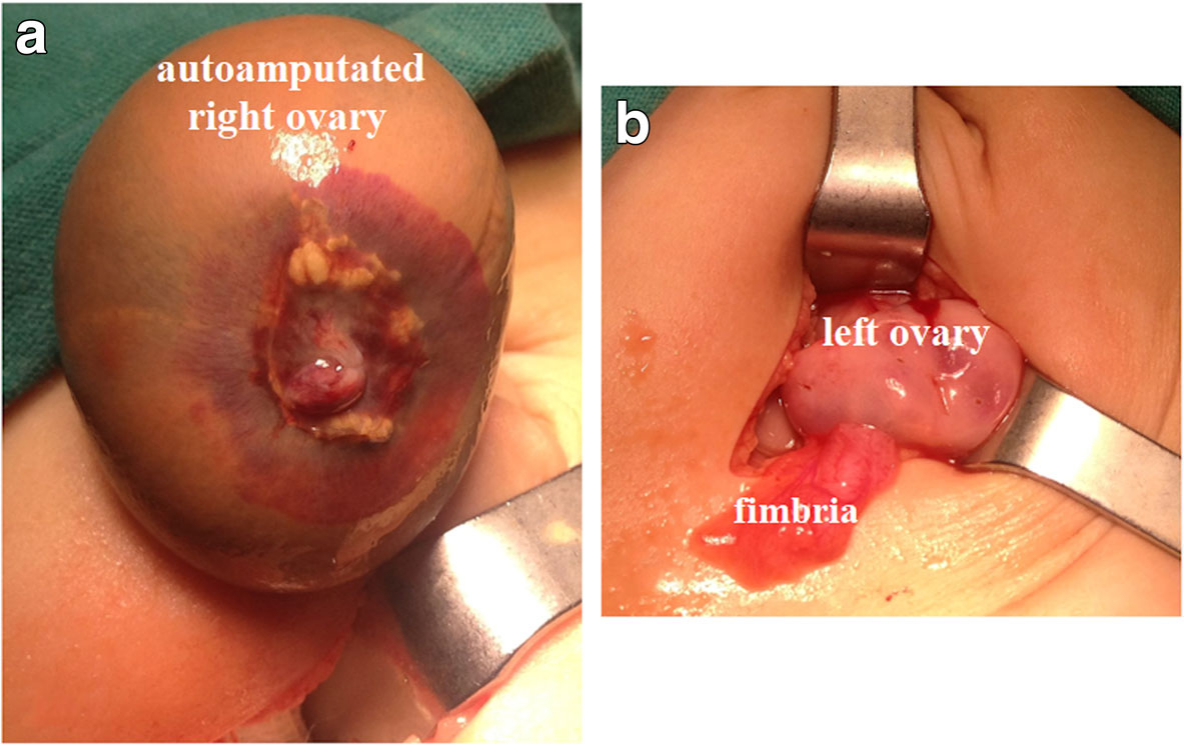
**a** Autoamputated wandering right ovary. **b** Operative view of the remaining left ovary with drained cysts and fimbria

A 55-day-old Caucasian baby girl was referred to us by a gynecologist who had detected an intraabdominal cystic mass during prenatal US in the last trimester and was normal upon physical examination; no palpable abdominal masses were found. No other factors were present in her history. Imaging studies showed intraabdominal cystic mass. The tumor markers that were examined to determine the presence of malignancy were within normal ranges. She underwent surgery; her right ovary was found to be autoamputated due to torsion. The amputated necrotic, wandering ovary was removed, and the cysts in the contralateral ovary were drained (Fig. 2a, b); an incidental appendectomy was performed. Pathologic examination of the patient confirmed the diagnosis of a necrotic ovary. She had cysts smaller than 1 cm, they were aspirated during surgery. Postoperatively the cysts redeveloped and spontaneously disappeared after 1 year of follow up. She developed no wound infections or peritonitis during the PO period and was discharged on the third PO day. After surgery, her ovary was checked regularly using pelvic and Doppler imaging. The development of her remaining ovary was age appropriate. She has been followed for 2.5 years, and she continues to be followed uneventfully.

### Case 3

**Fig. 3:**
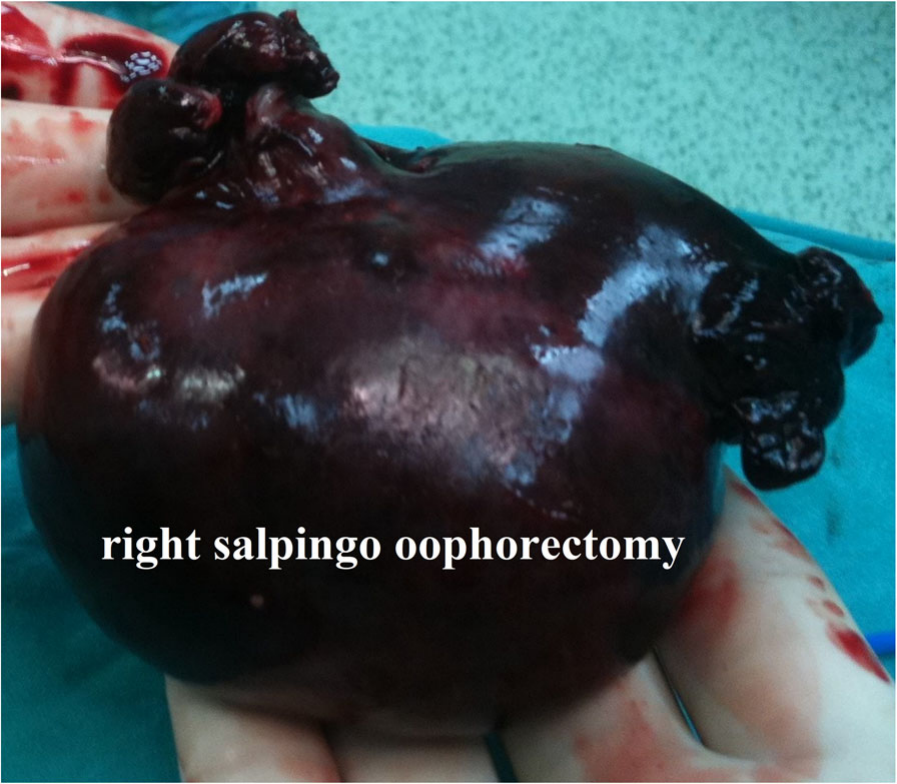
Specimen of the right salpingo-oophorectomy

An 8-year-old Caucasian girl was admitted to our hospital with acute abdominal symptoms 60 hours after the complaints started; serious acute pelvic pain, sudden onset of nausea, vomiting, and pelvic pain and tenderness were present. No other factors were reported in her history. Direct abdominal X-ray images were normal. She was pre-diagnosed as having appendicitis or ovarian pathology based on anamnesis, a physical examination, and laboratory findings. She was operated on under emergency conditions and without prior US investigation. On operation, torsion was detected in her right ovary. She had a necrotic right ovary and salpinx (Fig. 3); a salpingo-oophorectomy was performed due to the adnexa showing no improvement in its black color and necrotic appearance after detorsion. The contralateral ovary was normal, and an incidental appendectomy was performed. The pathology report indicated a hemorrhagic infarct in the ovary. She developed no wound infections or peritonitis during the PO period and was discharged on the fifth PO day. After surgery, her remaining ovary was examined regularly using pelvic and Doppler imaging. The development of her remaining ovary was also age appropriate. She has been followed for 6 years, and she continues to be followed uneventfully.

### Case 4

**Fig. 4:**
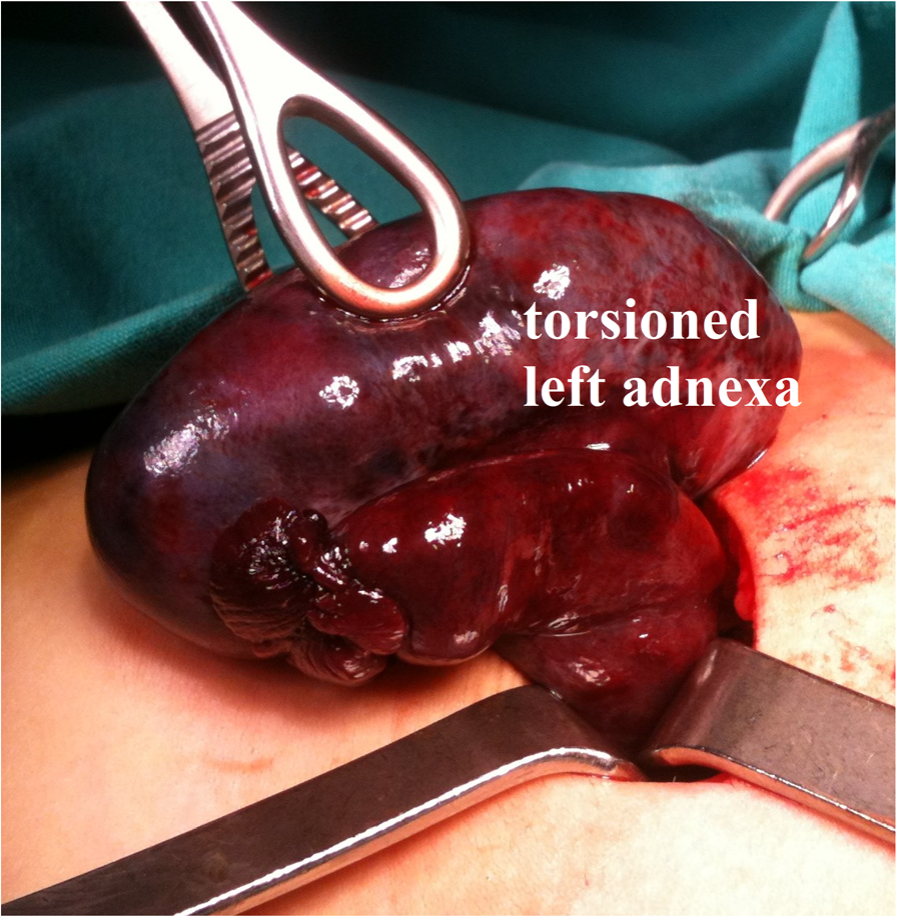
Operative view of the torsioned left adnexa

A 10-year-old Caucasian girl was admitted with serious acute pelvic pain 4 hours after the complaints started. Acute abdominal symptoms were present in the patient; sudden onset of nausea, vomiting, and pelvic pain and tenderness were reported. Direct abdominal X-ray images were normal. No other factors were present in her history. She was pre-diagnosed as having appendicitis or ovarian pathology. On exploration, left adnexal torsion was detected, and detorsion was performed (Fig. 4). The ligaments were extremely long; the ipsilateral mesosalpinx was shortened with a nonabsorbable suture, and an incidental appendectomy was performed. No wound infections or peritonitis developed during the PO period and she was discharged on the third PO day. After surgery, her ovaries were checked regularly using pelvic and Doppler imaging. In the second month, the affected ovary was similar in size to the contralateral ovary, and normal blood flow was observed on US. The development of her ovaries was also age appropriate. She has been followed for 6 years. She continues to be followed uneventfully and has regular menstrual cycles.

### Case 5

**Fig. 5:**
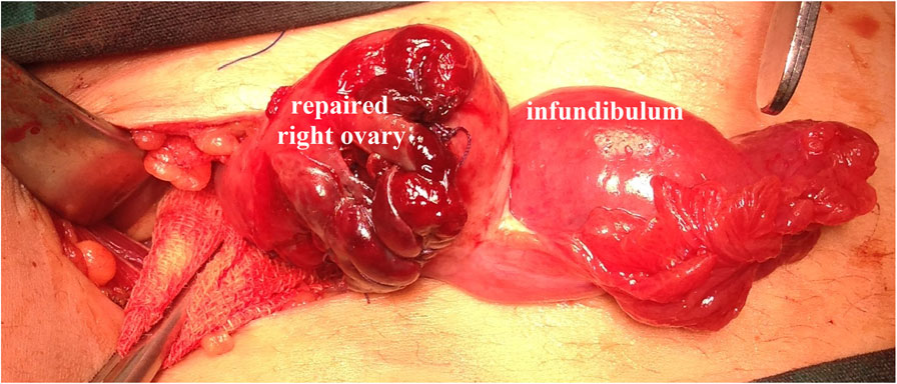
Repaired right ovary after detorsion and cystectomy. Enlarged and edematous fallopian tube

A 16-year-old Caucasian girl visited our hospital with serious acute pelvic pain 30 hours after her complaints started. Acute abdominal symptoms were present; sudden onset of nausea, vomiting, and pelvic pain and tenderness were reported. She had a normal menstrual cycle. Direct abdominal X-ray images were normal. No other factors were present in her history. The tumor markers that were examined to determine the presence of malignancy were within normal ranges. Preoperative abdominal US was performed; Minimal pelvic fluid, an increase in the diameter of her appendix, and a large right ovary with increased diameter relatively to the left ovary were found on US examination and torsion was suspected in her right ovary. During the operation, right ovarian torsion with a hemorrhagic cyst approximately 8 cm in diameter was detected. A cystectomy was performed to protect her ovary against retorsion, and her ovary was repaired (Fig. 5). The contralateral ovarian cysts were drained by aspiration. Her appendix was turgid and edematous and was evaluated as periappendicitis. In this case, as with the other four patients, an incidental appendectomy was performed. She developed no wound infections or peritonitis during the PO period and was discharged on the fifth PO day. After surgery, her ovaries were checked regularly using pelvic and Doppler imaging. In the fourth month, her affected ovary was similar in size to the contralateral ovary, and normal blood flow was observed on US. She has been followed for 1.5 years. She continues to be followed uneventfully and has regular menstrual cycles. The development of her ovaries was also age appropriate.

## Discussion

The main causes of non-neoplastic ovarian torsion are: enlargement of the ovaries due to a cyst; abnormally long fallopian tubes, mesosalpinx, and mesovarium; development of adnexal venous congestion due to constipation, sigmoid colon distension, pregnancy, premenarchal hormonal activity; and jarring movement of a relatively large ovary with an infantile uterus [4]. In the two newborns in this series (cases 1 and 2), cysts developed with hormonal activity during pregnancy, leading to torsion. In one of the three adolescents (case 3) who underwent salpingo-oophorectomy, no pathology that led to torsion could be detected. A long mesosalpinx caused ovarian torsion in case 4; a hemorrhagic cyst that developed after premenarchal hormonal activity may have led to torsion in case 5.

Ovarian torsions are difficult to diagnose not only preoperatively but also intraoperatively. A precise diagnosis of preoperative ovarian torsion is not always possible because imaging methods such as US, Doppler imaging US, CT, and magnetic resonance imaging (MRI) are not specific for ovarian torsions in children [7, 8]. During the operation, the surgeon subjectively decides whether the patient has ischemic ovaries via inspection, ordinarily based on ovarian color (blue, purple, black, black-bluish, purple-black, or ink black) [4, 5, 9]. Therefore, the ovary should detorsioned and monitored, and oophorectomy may be planned during a second procedure. Vascularity and normal follicular development have been demonstrated even in ovaries that appear ischemic [9, 10].

In recent years, because pelvic structures can be better evaluated due to the increased number of laparoscopic minimally invasive procedures [11], the numbers of oophoropexy and second-look cases have increased [1, 12]. However, if laparotomy is to be performed in acute cases with a preoperative diagnosis of ovarian pathology, a Pfannenstiel incision is preferred for better evaluation of both ovaries and ligaments as well as for appendectomy. Using this incision, better exploration was possible in the case 1, who exhibited a pelvic mass. Laparoscopy was not performed for the cases in this series due to technical reasons.

No accepted routine practice exists to prevent retorsion of the same and/or opposite ovary after detorsion or oophorectomy. Upper pole excision, cyst aspiration and cystectomy are the classic methods that have been used for many years [1, 13]. For prevention of retorsion, cystectomy was performed in one patient in this study, and cyst drainage was performed in three patients.

Two main methods are used for fixation of the ovary. The first method involves fixing the ovary to the adjacent tissues: to the posterior abdominal peritoneum between the ureter and the iliac veins, between the ureter and the mesorectum, to the posterior uterine serosa, to the round or uterosacral ligaments, or to the sidewall of the pelvis. The second method, used for patients who have long utero-ovarian ligaments, is to shorten the ligaments by plication [1, 14–18]. Abeş and Sarihan reported that ten patients who underwent laparotomy with ovarian torsion had remaining single or both ovaries fixed to the posterior abdominal peritoneum with an absorbable suture [18]. Ashwal *et al*. reported that 7 torsions recurred in 6 of 32 premenarchal patients who had been followed for 15 years and that even second and third recurrences could develop. Therefore, ovarian ligament plication were performed [1]. Fuchs *et al.* performed case-based fixation for torsion with different methods in eight patients (seven adults), including two patients for their first torsion and five patients for their second torsion; in the final patient, oophoropexy was performed twice for second and third torsions [16]. After combining their results with those reported in the literature, the authors concluded that ligament plication was easier and more secure than other methods [16]. Because recurrences have been reported when absorbable sutures are used to fix ovaries, nonabsorbable sutures are recommended regardless of the method of choice [16, 19].

Controversy exists regarding whether oophoropexy should be performed during the same session or during a second session, when edema and hemorrhage have disappeared and after malignancy is no longer suspected [4]. Contrary to the studies that advocate fixing the remaining single ovary during the same session, even if the appearance of the ovary is normal [2, 20–22], oophoropexy was reported by some studies only in recurrent cases [17, 19]. Childress and Dietrich favored ovary protection but noted that, because the efficacy and safety of ovarian fixation were not well-established, oophoropexy could be performed if only one ovary remained due to prior oophorectomy [13]. As an alternative method, Svensson *et al*. treated a torsion that developed on the contralateral side in a patient who had undergone a right salpingo-oophorectomy 2 years prior with detorsion-hyperbaric oxygen therapy followed by an oophoropexy 1 month later [14].

In an 11-case series, Comeau *et al.* reported that oophoropexy was evaluated on a case-by-case basis at the discretion of the senior surgeon, oophoropexy was performed for the first, second or third torsion [23]. The cases in this series were also evaluated individually. Oophoropexy was performed on patients who had only one remaining ovary by fixing the ovary to the pelvic sidewall in case 1 and by ligament fixation in case 4 due to excess mobility of the adnexa. In the third patient with only one ovary, oophoropexy was evaluated as an option but was not performed.

As Crouch *et al.* noted, unfortunately, ovarian torsions are often confused with appendicitis, which is the most common cause of acute abdomen in the childhood period; these patients undergo surgery without proper examination under emergency conditions [12]. The three adolescent patients (cases 3, 4, and 5) in this small series underwent surgery with the pre-diagnosis of acute abdomen. Their appendix was found to be inflamed due to the ovarian torsion, which was thought to be periappendicitis. However, appendectomy was performed in all five patients after considering the advantages and disadvantages of the procedure [24].

The long-term results of oophoropexy are unclear. All studies conducted on the advantages and disadvantages of the methods used have included a small number of patients. The main objections to oophoropexy are retorsion and periodic pelvic pain [17, 19], and mechanical infertility due to disruption of the anatomy of the uterus, ovary, and fallopian tubes in cases in which the ovary is fixed [8, 12]. The objections to the ligament plication technique are the breakdown of ovarian vascularity with the ligament adjacent to the ovarian artery, fallopian tube damage [14, 19], and the occurrence of ovarian atrophy following fixation [25]. In addition, experience with ovarian fixation has increased due to the use of protective ovarian transposition for patients who receive pelvic radiotherapy. Contrarily ovarian torsion developed in two adult patients who underwent oophoropexy to avoid pelvic radiation [26].

However, it is also recommended that the fixation process should be standardized, similar to testicular torsions [12, 18, 22]. Significant disadvantages of ovarian protection methods in the pediatric age group are difficulties determining exactly where to fix the ovary and what is the normal length of the ligament; because the anatomy of pelvic structures changes from birth to puberty, the ovarian fossa dimensions continue to grow throughout the premenarchal period. In addition, after menarche, the size and morphology of the ovary change with the menstrual cycle. When considering the question of why a standard process cannot be applied to ovarian torsions in the pediatric population as with orchiopexy in testicular torsions, the answer is manifold. First, multiple factors lead to ovarian torsions; ovaries are intraabdominal organs, and the physiology and anatomy of pelvic structures changes more with age in females than in males.

## Conclusions

A definitive diagnosis of ovarian torsion is made at the time of surgery. Therefore, the surgeon must have knowledge of detorsion and fixation techniques. Fixation of the ovaries during the same or a second operative session will be discussed more frequently as the number of ovary-protective open and laparoscopic cases increases. However, the long-term outcomes of ovary fixation cases will affect the implementation of this decision.

## Abbreviations

CT: Computed tomography
MRI: Magnetic resonance imaging
PO: Postoperative
US: Ultrasonography
